# The non-death role of metacaspase proteases

**DOI:** 10.3389/fonc.2012.00078

**Published:** 2012-07-24

**Authors:** Amit Shrestha, Lynn A. Megeney

**Affiliations:** ^1^Regenerative Medicine Program, Sprott Centre for Stem Cell Research, Ottawa Hospital Research Institute, The Ottawa Hospital,Ottawa, ON, Canada; ^2^Department of Cellular and Molecular Medicine, University of Ottawa,Ottawa, ON, Canada

**Keywords:** metacaspase, caspase, non-death, cell cycle, proteostasis

## Abstract

The activation of caspase proteases and the targeting of protein substrates act as key steps in the engagement and conduct of apoptosis/programmed cell death. However, the discovery of caspase involvement in diverse non-apoptotic cellular functions strongly suggests that these proteins may have evolved from a core behavior unrelated to the induction of cell death. The presence of similar proteases, termed metacaspases, in single cell organisms supports the contention that such proteins may have co-evolved or derived from a critical non-death function. Indeed, the benefit(s) for single cell life forms to retain proteins solely dedicated to self destruction would be countered by a strong selection pressure to curb or eliminate such processes. Examination of metacaspase biology provides evidence that these ancient protease forerunners of the caspase family also retain versatility in function, i.e., death and non-death cell functions. Here, we provide a critical review that highlights the non-death roles of metacaspases that have been described thus far, and the impact that these observations have for our understanding of the evolution and cellular utility of this protease family.

## INTRODUCTION

The conserved family of clan CD proteases, caspases, has been extensively characterized in programmed cell death or apoptosis, a function that is vital for homeostasis of complex organisms. Despite the well established death centric role, there is increasing evidence for caspase involvement in non-apoptotic scenarios, such as terminal differentiation of numerous cell types, non-death cellular remodeling events and immune system adaptation. In addition to the study of caspase function in multi-cellular life forms, the discovery of functional caspase orthologs in lower eukaryotes, such as fungi and protozoa (termed metacaspases) suggests these proteins emerged early in the evolutionary record (Uren et al., 2000; Aravind and Koonin, 2002).

Interestingly, recent investigations of metacaspase function have revealed these enzymes play a role in various non-apoptotic or “non-death” processes, in a manner analogous to the metazoan caspase family. Here, we critically review the literature and the latest studies which examine the physiologic function of metacaspase proteases. We conclude that the versatility displayed by the caspase protease family may simply reflect primordial death and non-death functions that initially evolved from para- and metacaspase activity, a functional diversity that is clearly present in unicellular organisms such as yeast and trypanosomatids.

## METACASPASES IN CELL CYCLE REGULATION

One of the earliest reports indicative of a non-death role for metacaspases was derived from observations in the protozoan *Trypanosoma brucei*. Here, the expression of several metacaspases (MCA2/3/5) was shown to be critical for the viability of the bloodstream form of the parasite. RNAi induced knockdown of the expression of these metacaspase genes was accompanied by severe growth retardation and cell cycle defects of the circulating *Trypanosoma* (Helms et al., 2006). Furthermore, the Δ*mca2/3*Δ*mca5* mutants showed no significant difference in cell death kinetics in response to prostaglandin D_2_ treatment, observations which support a cellular role for *Trypanosoma* metacaspase beyond the apoptosis cascade.

A subsequent study in the related kinetoplast protozoan, *Leishmania major*, further supported the role of metacaspases in cell cycle dynamics. The *L. major* metacaspase (LmjMCA), which is syntenic to MCA5 in *T. brucei*, was observed to be a critical component that regulated stage progression during cellular division (Ambit et al., 2008). For example, during logarithmic growth, LmjMCA expression increased when compared to stationary phase cultures. Moreover, the association of LmjMCA with mitotic spindles during cellular division provided convincing evidence that this metacaspase impacted cell cycle progression. Accordingly, the overexpression of LmjMCA resulted in severe growth retardation with concurrent defects in kinetoplast segregation, multiple mitotic nuclei, and changes in ploidy with a reduced number of cells undergoing cytokinesis. Attempts to create an LmjMCA null strain also resulted in striking cell cycle defects, leading to lethality. Together, these observations suggest that LmjMCA plays a critical role in the management of cell cycle progression.

The mechanism by which a protozoan metacaspase exerts cell cycle control is not entirely clear, although a number of studies suggest that the subcellular localization of the enzyme as well as the level of expression may dictate this non-death activity. First, the RAB11 marker for recycling endosomes was observed to co-localize with a large proportion of the metacaspases in a distinctive compartment between the nucleus and the kinetoplast (Helms et al., 2006). However, the recycling process of VSG was observed to be independent of the metacaspases. RAB11 positive endosomes are known to be involved in kinetoplast division leading to cytokinesis in the procyclic form of *T. brucei* (Jeffries et al., 2001; Kohl et al., 2003). Thus, the role of these metacaspases in cytokinesis of the bloodstream form of *T. brucei* may argue a non-death role in cell cycle progression, yet specific experiments to support this contention have yet to be undertaken. More recently, overexpression of the *Trypanosoma cruzi TcMCA3* has been linked to a non-death biologic activity, resulting in a reduced growth rate and a transient G1/S block. Additionally, overexpression of TcMCA5 lacking the Ct region (pro, gln, and tyr rich region) led to increase in hypodiploid cells, which implicates the Ct region in dictating metacaspase function (Laverriè, 2012). Of note, MCA5 is syntenic in the three protozoa species (Mottram et al., 2003); however, the ability of the Ct region to mediate metacaspase function has yet to be explored in *L. major* and *T. brucei*. Together, these observations in related protozoa species argue that metacaspases regulate cell cycle progression, a function that appears to be independent of promoting cell death.

The metacaspase involvement in cell cycle control appears to be a well conserved phenomenon that extends across phyla. In *Saccharomyces cerevisiae*, deleting the single metacaspase Yca1 (Δ*yca1*) or altering the proteolytic activity of the enzyme leads to altered DNA content and growth rate, which is marked by a slowed G1/S transition (Lee et al., 2008). A similar trend has also been reported for *T. cruzi* (Laverrière, 2012). In addition, yca1 null cells failed to respond to a nocodazole-induced mitotic G2/M checkpoint in conditions that favored cell growth. Taken together, these observations implicate Yca1 in regulation of cell cycle checkpoints. Similarly, the metacaspase of the related yeast species, *Schizosaccharomyces pombe* also impacts cell cycle dynamics. In this instance, overexpression of the fission yeast metacaspase, Pca1, led to accelerated growth, a feature which was much improved upon cadmium induced oxidative stress (Lim et al., 2007). The precise mechanism by which a metacaspase protease regulates cell cycle progression remains unknown yet is of considerable interest.

## METACASPASE REGULATION OF PROTEOSTASIS AND PROTEIN AGGREGATE FORMATION

The ability of the pombe metacaspase to promote cell cycle advance during oxidative stress strongly suggests that this clade of enzymes may have a cytoprotective role, a feature that appears contrary to the well-described death centric behavior described to date. Consequently, in a subsequent study in *S. cerevisiae* we identified the regulation of protein aggregates as a function by which Yca1 may confer improved fitness and survival (Lee et al., 2010). A genome wide proteomic analysis showed that Δ*yca1* cells are enriched for the Hsp70 family of chaperones (Ssa1, Ssa2, and Ssa4) as well as Hsp104 remodeling chaperone that is involved in the disaggregation of insoluble protein aggregates (Parsell et al., 1994). Furthermore, the normally cytosolic YCA1-GFP was observed to co-localize with Hsp104-RFP, a marker for protein aggregates, under heat stress independent of its catalytic activity. Consequently, filter-trap analyses showed that the loss of Yca1 or its catalytic activity was synonymous with increased levels of insoluble protein aggregates (Lee et al., 2010). Truncated forms of Yca1 lacking the polyQ region were observed to shift from the insoluble protein fraction to a more equitable distribution, with the truncated Yca1 contained in both the soluble and insoluble protein fractions. These observations would suggest that the polyQ region is responsible (in part) for the targeting of Yca1 to aggregated material/proteins and that the stability and/or dissipation of protein aggregates are controlled by the yeast metacaspase Yca1. This unexpected feature of Yca1 appears to be independent of invoking cell death and is associated with maintaining proper cell cycle progression.

As noted with cell cycle regulation and metacaspase activity, the apparent role of a metacaspase(s) in regulating protein levels may also be a conserved molecular function for this otherwise death centric protein. In support of this contention, a study in the filamentous fungus, *Aspergillus fumigatus* revealed that loss of metacaspase expression led to a blunted response to endoplasmic stress (ER) induction (Richie et al., 2007). Specifically, the induction of ER stress in cells lacking functional metacaspase using 2-deoxy-D-glucose (2-DG), tunicamycin (TM), and dithiothreitol (DTT) displayed retardation in growth. Moreover, the increased sensitivity to the glucose analog, 2-DG induced stress in Δ*casA*/Δ*casB* cells was particularly highlighted in the study. 2-DG is known to induce the unfolded protein response (UPR) which delays protein synthesis in order to allow for either the successful re-folding or degradation of misfolded proteins to ensure ER homeostasis (Wu and Kaufman, 2006). In addition to regulating protein homeostasis, these authors also observed that apoptosis induction proceeded independent of metacaspase activity. For example, there was no significant change in the number of PI-positive protoplasts, an observation that is strikingly similar to the observations of metacaspase independent cell death that have been reported in *S. cerevisiae* (Madeo et al., 2009). Although not definitive, the protein homeostatic behavior attributed to metacaspases in yeast and fungi species in the above mentioned studies imply an ancient non-death regulatory role for these enzymes.

Given the observations above it is tempting to speculate that all caspase/metacaspase enzymes have evolved a proteostasis function. Nevertheless, a separate study in *A. nidulans* suggests that unlike in *A. fumigatus*, where loss of both metacaspases had an additive impact on stress outcomes, the metacaspases in *A. nidulans* may actually retain inhibitory or antagonistic functions related to maintaining protein stability (Colabardini et al., 2010; Tsiatsiani et al., 2011). Here, ER stress was induced by treating cells with farnesol, which is also known to induce the UPR, as well as DTT and 2-DG. Spotting assays with the different treatments showed that the loss of *cas*B had a much more significant effect on growth in comparison to the Δ*cas*A cells. Overexpression of pkcA in Δ*cas*A cells restored the sensitivity to farnesol-induced apoptosis. These observations led the authors to speculate that in *A. nidulans* metacaspases may function antagonistically with *cas*A promoting death while *cas*B has a protective role during ER stress (Colabardini et al., 2010).

## FUNCTIONAL OVERLAP BETWEEN CASPASES AND METACASPASES

**FIGURE 1:**
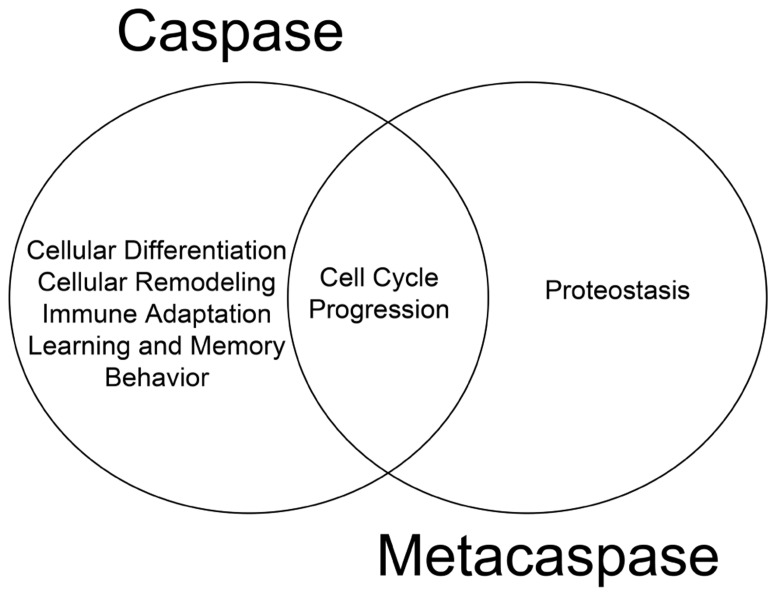
**Functional overlap of caspase and metacaspase function**. Regulation of cell cycle progression is a conserved function for both proteases, suggesting a common evolutionary origin. Additional non-death functions of metacaspases such as the role of Yca1 in proteostasis has yet to be confirmed as a feature of caspase biology.

The cellular behavior of metacaspases described thus far provides substantial evidence that these proteases are physiologically active and retain critical function(s) independent of apoptosis. We have previously argued that death and differentiation may be of a common origin and that different stimuli/substrates may dictate the outcome (Fernando and Megeney, 2007). The physiological functions of metacaspases discussed thus far which are mirrored by their metazoan counterparts lend further support to the hypothesis (**Figure 1**). For example, the ability of metacaspases to regulate cell cycle events have also been observed for their metazoan counterparts; human hepatoma cells that lack caspase 3 activity have also been shown to bypass the G2/M mitotic checkpoint in response to nocodazole treatment (Hsu et al., 2006) and thus implicating an evolutionarily conserved function for caspase and metacaspases between different phylogeny (Tsiatsiani et al., 2011). To date, it remains unclear whether metacaspases have the ability to alter cell fate in a manner similar to metazoan caspases. With reference to mammalian caspase enzymes, up-regulation of caspase 3 activity is a critical step in promoting cell differentiation in virtually all progenitor cell types examined from skeletal muscle, to neurons, hematopoietic cell lineages and ES cells (Fernando et al., 2002, 2005; Fujita et al., 2008; Janzen et al., 2008). Moreover, the role of caspase 3 in determining cellular differentiation is conserved across the phyla, from *Drosophila* to humans (Abdul-Ghani and Megeney, 2008).

The importance of caspase 8 activity for trophoblast fusion during human placental development (Black et al., 2004) and caspase 9 as an initiator of lens fiber epithelial development (Weber and Menko, 2005) suggests that both initiator and executioner caspase enzymes have the ability to function in non-death scenarios. The metacaspases involved in non-death functions described here thus far belong to or are known to resemble the type I category, which is thought to be similar to the initiator or pro-caspases in metazoans; both sets possess a regulatory region in the N-terminal. As for type II metacaspases, which are predominantly present in plants (Tsiatsiani et al., 2011), they have yet to be reported in processes other than cell death. Given these observations it is tempting to speculate that the non-death function of initiator caspases may have evolved from the non-death targeting activity of type I metacaspase enzymes.

The role of metacaspases in maintaining protein homeostasis is a more recent discovery that is unique to *S. cerevisiae* and has yet to be explored extensively in other organisms. Nonetheless, the novel findings generated from the yeast studies provide support for the postulation that Yca1 regulation of protein aggregates may be a mechanism by which the cell increases fitness and adaptation to stress (Lee et al., 2008, 2010). The beneficial role of Yca1 in proteostasis is largely in contrast to the negative role ascribed to mammalian caspases in the same context. Here, caspase proteases have garnered considerable interest for as causative agents in various neurodegenerative/neuromuscular disease conditions such as Alzheimer’s disease, Huntington’s disease, Parkinson’s disease, amyotrophic lateral sclerosis (ALS), and inclusion body myopathies. In these instances, caspase activation is believed to contribute to the development of a proteotoxic environment by cleaving various proteins that in turn promote aggregate formation, leading to cell stress and eventual cell death (Rothstein, 2009; Rohn, 2010; Graham et al., 2011).

**FIGURE 2:**
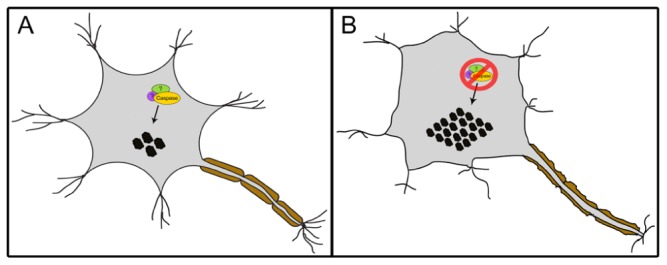
**A proposed model of a caspase-dependent mechanism involved in maintaining proteostasis**. The interaction of caspases, possibly with yet unidentified additional cofactors, facilitates the regulation of protein aggregates (black circles) within mammalian cells such as neurons **(A)**. The loss of this mechanism leads to increase in aggregate levels resulting in stress **(B)**.

In contrast to the well-accepted contention that activated caspases are synonymous with deleterious activity in neurons, a number of studies suggest that caspase activity may be required for neural cell adaptation and may counteract proteotoxicity. First, caspase activation has been shown to mediate long-term potentiation, learning, dendrite, and axon remodeling, all of which are independent of cell death (Huesmann and Clayton, 2006; Fernando and Megeney, 2007; D’Amelio et al., 2010; Li et al., 2010). More recently, caspase 3 has been reported to cleave TDP-43 in mouse primary cortical neurons, a response which attenuates TDP-43-induced apoptosis (Suzuki et al., 2011). Abnormal aggregated forms of hyperphosphorylated TDP-43 are the major components of ubiquitinated inclusion bodies (IBs) that characterize ALS and frontotemporal lobar degeneration with ubiquitinated inclusions (FTLD-U; Neumann et al., 2006). The study of Suzuki et al. (2011) demonstrated that ER stress or staurosporine treatment led to caspase cleavage of TDP-43 and generation of C-terminal fragments (CTFs). The death inducing ability of the resulting CTF aggregates were lower than the wildtype TDP-43, and of particular note a caspase cleavage resistant mutant of TDP-43 showed a magnified death response compared to the wildtype protein; an observation that emphasizes a cytoprotective response of the caspase cleavage event (Suzuki et al., 2011). Contrary to the study by Suzuki et al. (2011), other groups have shown that CTFs of TDP-43 can itself be toxic and induce cell death (Johnson et al., 2008; Zhang et al., 2009 However, the cell death in these latter studies may be simply a reflection of a caspase activation pattern that is unrestrained and is coincident with the TDP-43 modifications, rather than disease causing *per se*. Indeed, a reasonable supposition may be that the caspase activation that accompanies protein aggregation in neurodegenerative disease conditions is an adaptive response to rid the cell of toxic materials rather than a disease propagating alteration (**Figure 2**). The corollary to this model would suggest that caspase mediated cell death ensues from excess activation of an otherwise beneficial response.

Interestingly, a recent structural comparison between the *T. brucei* metacaspase, MCA2 and caspase 7 suggests that despite overall structural similarity, metacaspases and caspases differ in their internal design (McLuskey et al., 2012). In addition, both proteases contain specific residues that facilitate substrate binding to the S1 pocket. Albeit these residues are not conserved between the proteases, the authors suggest that proteases within this family may share a common mechanism for substrate recognition. However, the Y31 residue involved in substrate binding and recognition is only conserved in *T. brucei* metacaspases (McLuskey et al., 2012). Thus, future structural analyses of other metacaspases are required to determine whether all metacaspases contain an amino acid residue equivalent to the Y31 residue within their N-terminal region. Of note, caspase 7 lacks an equivalent N-terminal region that is present in MCA2 and thus a comparison to initiator or pro-caspases such as caspases 8 or 10 may be favorable. Nonetheless, the structural similarities exhibited in this study reinforce the functional overlap between metacaspases and caspases in both death and non-death scenarios and further support the argument that both classes of proteases are evolutionarily conserved. Overall, the current evidence presented here suggests that metacaspase proteins act beyond their well described role in apoptosis. The non-death activity of metacaspase proteases reflect an ancient and conserved function that appears to extend to metazoan caspases, and may well represent the evolutionary origin of the death and non-death roles for these same proteins. Future studies to address proteostasis activity in the metazoan caspase family will be a critical step to evaluate and support such a hypothesis.

## Conflict of Interest Statement

The authors declare that the research was conducted in the absence of any commercial or financial relationships that could be construed as a potential conflict of interest.
